# Identification and characterization of new designer drug 4-fluoro-PV9 and α-PHP in the seized materials

**DOI:** 10.1007/s11419-015-0295-4

**Published:** 2015-10-13

**Authors:** Milena Majchrzak, Marcin Rojkiewicz, Rafał Celiński, Piotr Kuś, Mieczysław Sajewicz

**Affiliations:** Department of General Chemistry and Chromatography, Institute of Chemistry, University of Silesia, 9 Szkolna Street, 40-006 Katowice, Poland; Department of Organic Synthesis, Institute of Chemistry, University of Silesia, 9 Szkolna Street, 40-006 Katowice, Poland; Toxicology Laboratory ToxLab, 6 Kossutha Street, 40-844 Katowice, Poland

**Keywords:** Designer drugs, 4-Fluoro-PV9, α-PHP, ESI-MS/MS, GC–MS, NMR

## Abstract

In this study, we present identification and physicochemical characterization of new cathinone derivatives, 4-fluoro-PV9 and already known α-PHP in seized materials. Although the disclosure of α-PHP from an illegal product had been reported and characterized to some extent, the data on α-PHP are also presented together with those of 4-fluoro-PV9. The data of characterization for the two compounds were obtained by high-performance liquid chromatography (HPLC)–mass spectrometry and HPLC–diode array detection, electrospray ionization/ion trap mass spectrometry in MS^2^ and MS^3^ modes, gas chromatography–mass spectrometry, thermogravimetric analysis, differential scanning calorimetry, Fourier transform infrared spectroscopy, ultraviolet-visible spectroscopy, and nuclear magnetic resonance spectroscopy. To our knowledge, this is the first report for identification and detailed characterization of 4-fluoro-PV9 circulated on the illegal drug market.

## Introduction

In the global market of designer drugs, new products continuously appear that contain already known psychoactive compounds and mixtures thereof, but also new compounds, not yet characterized in any way [1–3]. According to the European Early Warning System, in Europe in 2005 alone, 14 novel psychoactive compounds were identified, and in 2013 the number of such compounds grew to over 80 items [1, 4]. Striking features are a wide spectrum of the marketed compounds and the forms in which they are traded. In Poland, consumables containing psychoactive ingredients are currently marketed as “fireplace kindling”, “bidet refreshers”, “humidity adsorbents”, “driver’s charms”, or “leech tamers”. Among the substances seized on the Polish and European Union drug market, derivatives of piperazine and phenylethylamine are frequently found, along with the synthetic cannabinoids, but the most frequently identified group is that of the cathinone derivatives [1–3, 5–11].

Synthetic analogs of cathinone can be divided into four groups; i.e., those containing *N*-alkyl- or alkyl/halogen substituents in any given position of the aromatic ring, those with methylenedioxy-substituted structures also in any given position of the aromatic ring, those with a pyrrolidinyl group at the nitrogen atom, and those with combined substituents of the second and the third groups [12–16]. Among the most frequently identified cathinones distributed on the Polish market, one can find 3-MMC (2-(methylamino)-1-(3-methylphenyl)-1-propanone), pentedrone (2-(methylamino)-1-phenyl-1-pentanone), 4-MEC (2-(ethylamino)-1-(4-methylphenyl)-1-propanone), MDPV (3,4-methylenodioxypyrovalerone), α-PVP (1-phenyl-2-(1-pyrrolidinyl)-1-pentanone), and MDPBP (3,4-methylenodioxy-α-pyrrolidinobutiophenone) [17–21].

Due to an increasing number of designer drugs available on the drug market and taking into account the vital possibility of synthesizing and manufacturing structural modifications of already known psychoactive substances, the availability of analytical characteristics of these compounds is inevitable for their efficient and rapid identification [8, 18, 22–26]. In this study, we present, for the first time to our knowledge, analytical characteristics of 4-fluoro-PV9 (1-(4-fluorophenyl)-2-(pyrrolidin-1-yl)octan-1-one) in seized materials. Although we could detect α-PHP (1-phenyl-2-(1-pyrrolidinyl)-1-hexanone) from another seized material, its disclosure from an illegal product had been reported by Uchiyama et al. [27].

We mainly present and discuss relevant analytical data obtained for 4-fluoro-PV9 and α-PHP by high-performance liquid chromatography–mass spectrometry (HPLC–MS) and high-performance liquid chromatography–diode array detection (HPLC–DAD), ion trap mass spectrometry with electrospray ionization (ESI) in the MS^2^ and MS^3^ modes, gas chromatography–mass spectrometry (GC–MS), thermogravimetric analysis (TGA), differential scanning calorimetry (DSC), Fourier transform infrared (FTIR) spectroscopy, ultraviolet-visible (UV-VIS) spectroscopy and proton and carbon nuclear magnetic resonance spectroscopy (^1^H NMR and ^13^C NMR).

## Materials and methods

### Chemicals

In our study, all reagents used were of the HPLC and MS purity grade. Water (Chromasolv), methanol, acetonitrile, 0.1 M formic acid and ammonium formate were purchased from Sigma-Aldrich, Poznań, Poland. For the NMR analysis, deuterated chloroform (CDCl_3_), also purchased from Sigma-Aldrich, was used.

### Sample preparation

The analyzed samples were seized by the police in 2014 in the Silesia region of South Poland and delivered to our laboratory for toxicological examination. Both samples were obtained in powdered form. Sample no. 1 was crumbled pale pink powder, and sample no. 2 was lumped beige powder.

For the purpose of liquid and gas chromatography, 10-mg aliquots of each powder were weighted, dissolved in 1 mL of acetonitrile/methanol (50:50, *v*/*v*) mixture, and ultrasonicated for 10 min. From each solution, 10 µL were collected and diluted with 990 µL methanol. Finally, the extracts were ready for analysis. For NMR spectroscopic analysis, 10 mg of each sample was dissolved in 0.6 mL CDCl_3_ and analyzed. For the purpose of FTIR, a 2-mg aliquot of each sample was taken and mixed with KBr, and pellets were formed. The KBr pellets were analyzed. For the purpose of TGA, DSC and UV-VIS spectroscopy, a 5-mg aliquot of each sample was taken for analysis. The TGA and DSC analysis were performed without any further sample treatment, and for the UV-VIS analysis, the samples were dissolved in methanol.

### HPLC–MS and HPLC–DAD conditions

In our investigations, the high-performance liquid chromatograph Thermo Surveyor coupled with the mass spectrometer Thermo LCQ DecaXP-Plus with an electrospray ionization source (Thermo Scientific, Warsaw, Poland) was used. The obtained data were processed with use of the Xcalibur and LCQTune programs (Thermo Scientific).

For the HPLC–MS analysis, the Hypersil RP C18 column (150 × 4.6 mm) (Thermo Scientific) was used and analysis was carried out in gradient mode. Mobile phase was composed of solvent A (0.02 M water solution of formic acid/0.05 M water solution of ammonium formate) and solvent B (10 % solvent A/90 % acetonitrile), and the following gradient program was applied: 0–2 min, 95 % (A) + 5 % (B); 2–30 min, 30 % (A) + 70 % (B); 30–32 min, 30 % (A) + 70 % (B); 32–40 min, the solvent ratio was returned to the initial state, i.e., 95 % (A) + 5 % (B). The mobile phase flow rate was 100 µL min^−1^.

The analytes were electrosprayed in the positive mode (ESI(+)-MS). Fragmentation in the ESI-MS^2^ and ESI-MS^3^ mode was carried out in the scanning range of *m/z* 50–500. The source temperature was 250 °C, and the carrier and ionizing gases were nitrogen and helium, respectively.

### GC–MS analysis

For GC–MS analysis, the Thermo Trace Ultra chromatograph was used, coupled with the Thermo DSQ mass spectrometer (Thermo Scientific). The analyses were carried out with use of the Rxi^®^-5Sil MS column (Restek, Bellefonte, PA, USA). The following working parameters were employed: injector temperature, 260 °C; oven temperatures, 100 °C for 2 min, ramp at 20 °C min^−1^ to 260 °C; carrier gas (helium) flow rate, 1.2 mL min^−1^; MS transfer line temperature, 250 °C; MS source temperature, 250 °C; injection volume, 1 μL, on splitless mode.

The obtained electron ionization (EI) mass spectra were compared to those of an EI-MS library [28] for structural confirmation.

### NMR spectroscopy

The NMR spectra were recorded with use of the UltraShield 400 MHz apparatus (Bruker, Bremen, Germany). Deutered chloroform was used as solvent.

### Thermogravimetric analysis (TGA) and differential scanning calorimetry (DSC)

TGA was carried out with use of the TGA/DSC1 Mettler–Toledo thermal analyzer (Mettler–Toledo, Greifensee, Switzerland), with a heating rate of 10 °C/min in a stream of nitrogen (60 cm^3^ min^−1^). DSC was performed with a TA-DSC 2010 apparatus (TA Instruments, New Castle, DE, USA) under nitrogen using aluminum sample pans. The DSC experiments were carried out in a nitrogen atmosphere with a temperature range from −70 °C to over the clearing point.

### Fourier transform infrared and UV-VIS spectroscopy

The infrared (IR) spectra were acquired with use of the PerkinElmer Spectrum One spectrometer (PerkinElmer, Waltham, MA, USA) using the KBr pellets. The UV-VIS absorption spectra were recorded in the methanol solution using the PerkinElmer Lambda Bio 40 UV-VIS spectrometer.

## Results and discussion

### Identification of the compound contained in sample no. 1

**Fig. 1:**
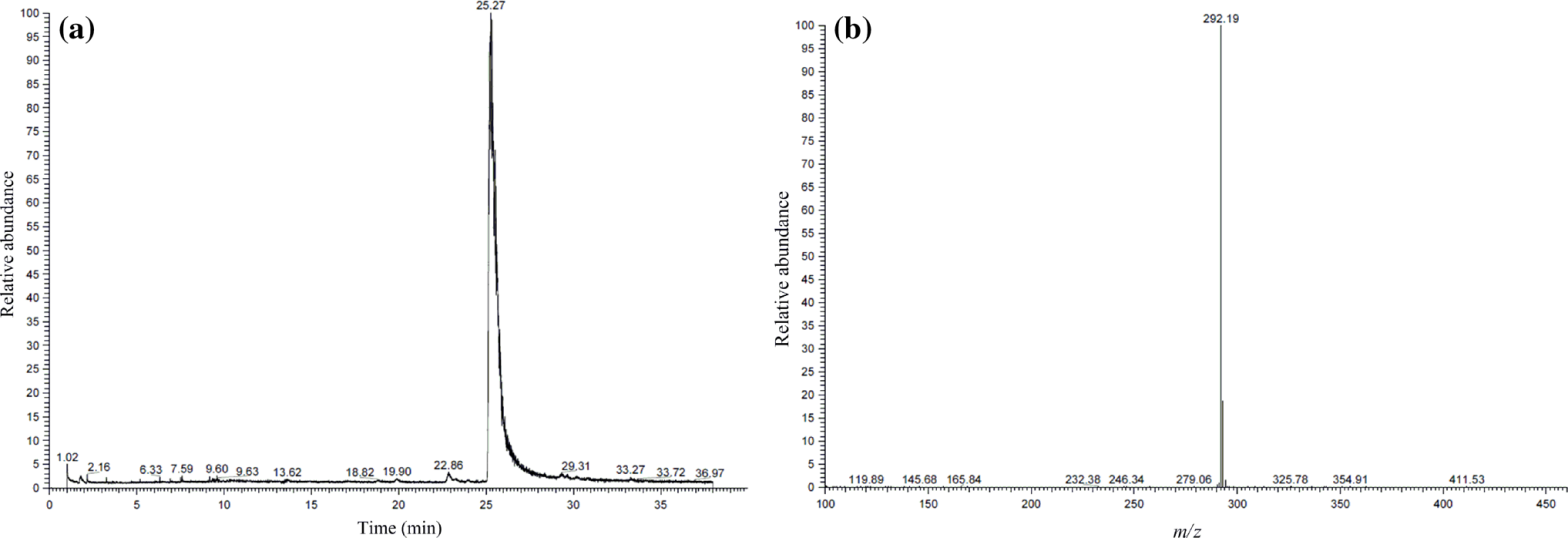
**a** Total ion current chromatogram (TIC) and **b** the single-stage mass spectrum of the intense peak appearing in the TIC obtained from the compound in sample no. 1, recorded by high-performance liquid chromatography–mass spectrometry (HPLC–MS)

**Fig. 2:**
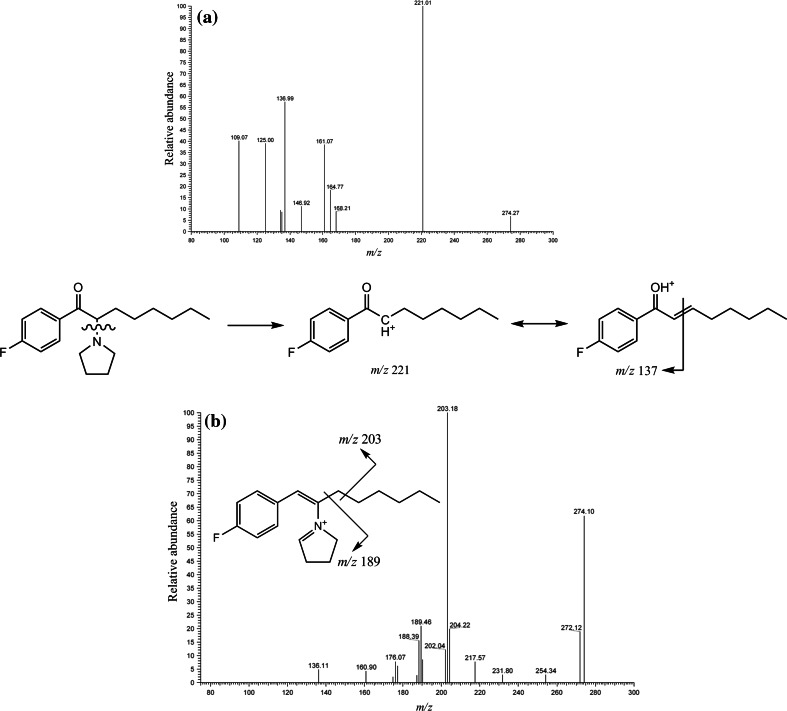
Product ion mass spectra obtained by ion trap mass spectrometry (MS) in the **a** MS^2^ (tandem) and **b** MS^3^ modes for sample no. 1. The precursor ions used for the MS^2^ and MS^3^ modes were those at *m/z* 292 and 274, respectively

Upon analysis of sample no. 1 by HPLC–MS, the total ion current (TIC) chromatogram (Fig. 1a) showed an intense peak at 25.27 min. Otherwise, no marked peaks appeared, showing that sample no. 1 contained almost a single compound with high purity (probably more than 95 %). Figure 1b shows the HPLC–MS spectrum of the intense peak observed in Fig. 1a; a base peak appeared at *m/z* 292. Because this peak seemed to be protonated molecular one [M + H^+^], the unknown compound may have a molecular weight of 291 Da. In the product ion mass spectra in the MS^2^ (tandem) mode using the peak at *m/z* 292 as the precursor ion, a peak at *m/z* 274 appeared (Fig. 2a), which indicated the elimination of one water molecule from the protonated molecular ion [M + H – H_2_O]^+^. This transformation is a well-known characteristic of cathinone derivatives. Given that this compound is assumed to be one of the cathinone derivatives, the product ion mass spectrum in the MS^3^ mode using the peak at *m/z* 274 as the precursor ion, which neither includes a carbonyl nor hydroxyl group, showed a base peak at *m/z* 203 and a fragment peak at *m/z* 189 (Fig. 2b), suggesting that the cathinone derivative has pyrrolidinyl and fluorophenyl moieties in its structure. The appearance of the base peak at *m/z* 221 in Fig. 2a is easily explicable by removal of the pyrrolidinyl moiety from the molecule.

**Fig. 3:**
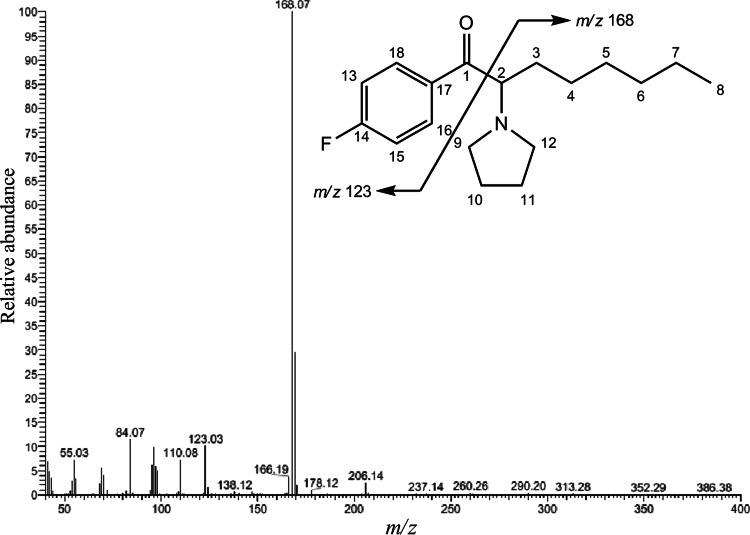
Mass spectrum of the compound contained in sample no. 1, obtained by gas chromatography–mass spectrometry (GC–MS) in the electron ionization (EI) mode

Unfortunately, in this study, we did not use the high-resolution MS that can give a molecular formula. Instead, we compared the GC–EI-MS spectra to those of an EI-MS spectral library, and fortunately, the EI-MS spectrum obtained from sample no. 1 matched well with that of 4-fluoro-PV9, which had very recently been added to the library. In both spectra, the peak at *m/z* 168 appeared as the base peak; the fragment peaks at *m/z* 169, 123, 110, 95, 84 and 55 appeared to be in common (Fig. 3).

Therefore, it seemed correct that the compound contained in sample no. 1 was fluoro-PV9, but the question of which position of the phenyl ring the fluorine atom was attached remained unclear, because it is well known that such regioisomers give the same or very similar EI-mass spectra.

**Fig. 4:**
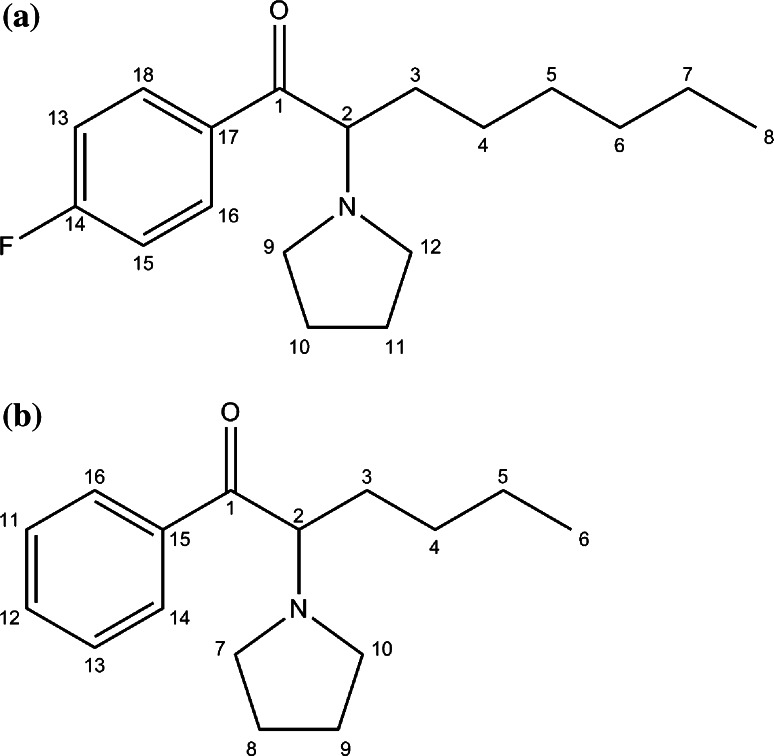
Target compound structures with carbon atom numbering for nuclear magnetic resonance analysis. **a** The compound contained in the sample no. 1; **b** the compound contained in sample no. 2

To finally determine the position of the fluorine atom in the aromatic ring, the NMR spectrum was recorded, as shown in Table 1. The carbon numbering is shown in Fig. 4a.

**Table 1:** ^1^H and ^13^C nuclear magnetic resonance (NMR) data for sample no. 1

Carbon atom position	Carbon chemical shifts (ppm)	Proton chemical shifts (ppm)
1	195.20	–
2	64.87	5.50 (dt, 1H)
3	31.13	2.08 (m, 2H)
4, 5, 6, 7	30.82; 29.11; 25.52; 22.36	1.15–1.35 (m, 8H)
8	13.85	0.79 (t, 3H)
9	52.72	3.12 (m, 1H); 3.86 (m, 1H)
10, 11	23.85; 23.66	2.18 (m, 4H)
12	51.23	3.69 (m, 2H)
13, 15	116.51; 116.53	7.22 (m, 2H)
14	165.53; 168.10	–
16, 18	131.88; 131.93; 131.96	8.13 (m, 2H)
17	131.79	–

The carbon atom numbering is shown in Fig. 4a

As a result, confirmation of the anticipated structure was obtained. Namely, in the aromatic range of the ^1^H NMR spectrum, two signal groups appeared, and witnessed to the presence of the fluorine atom at position *para* of the phenyl ring. Due to the proton interactions with the fluorine atom, two multiplets were observed instead of a “classical” signal arrangement (i.e., the doublets for each group of equivalent protons). Coupling with the fluorine atom was also visible in the ^13^C NMR spectrum, where signals originating from carbon atom no. 14, and those from no. 13 and 15 are split to the doublets. Due to the fact that sample no. 1 appeared as a hydrochloride, in the ^1^H NMR spectrum, a signal is observed at 11.95 ppm, originating from the proton of the quaternary pyrrolidine group. Therefore, it can be concluded that the compound contained in sample no. 1 is 4-fluoro-PV9 (1-(4-fluorophenyl)-2-(pyrrolidin-1-yl)octan-1-one). To our knowledge, this is the first report to identify 4-fluoro-PV9 in an authentic seizure sample.

### Identification of the compound contained in sample no. 2

**Fig. 5:**
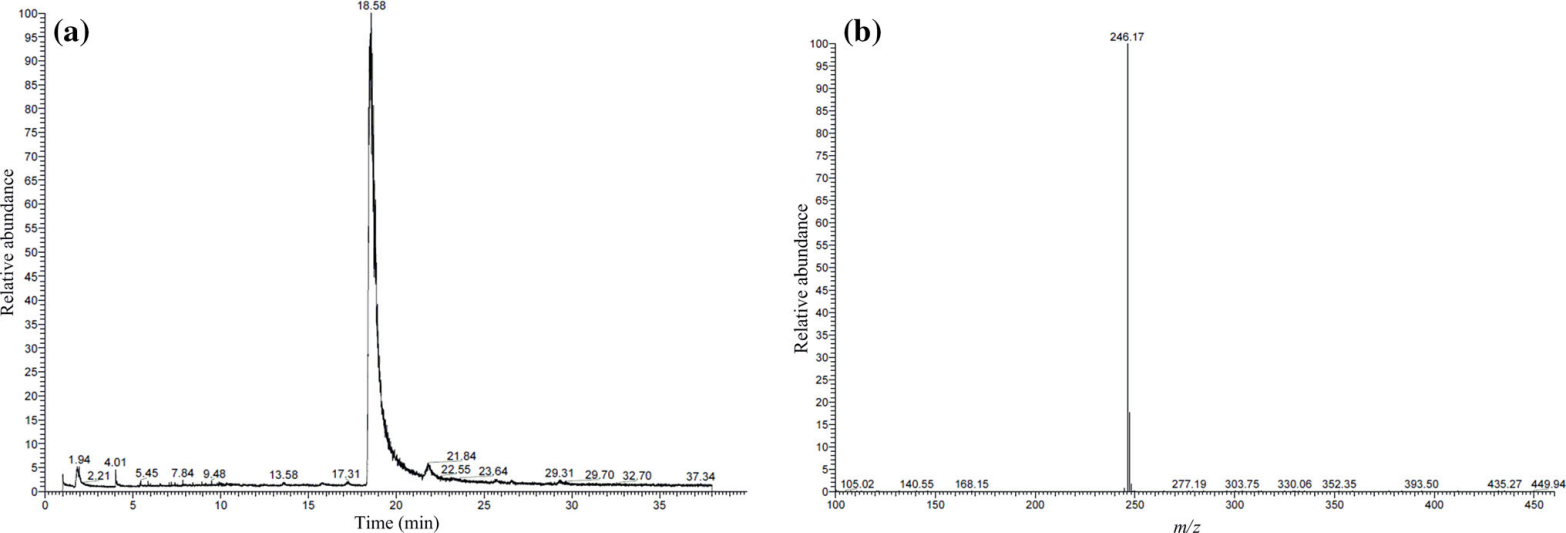
**a** TIC and **b** the single-stage mass spectrum of the intense peak appearing in the TIC, obtained from the compound in sample no. 2 recorded by HPLC–MS

**Fig. 6:**
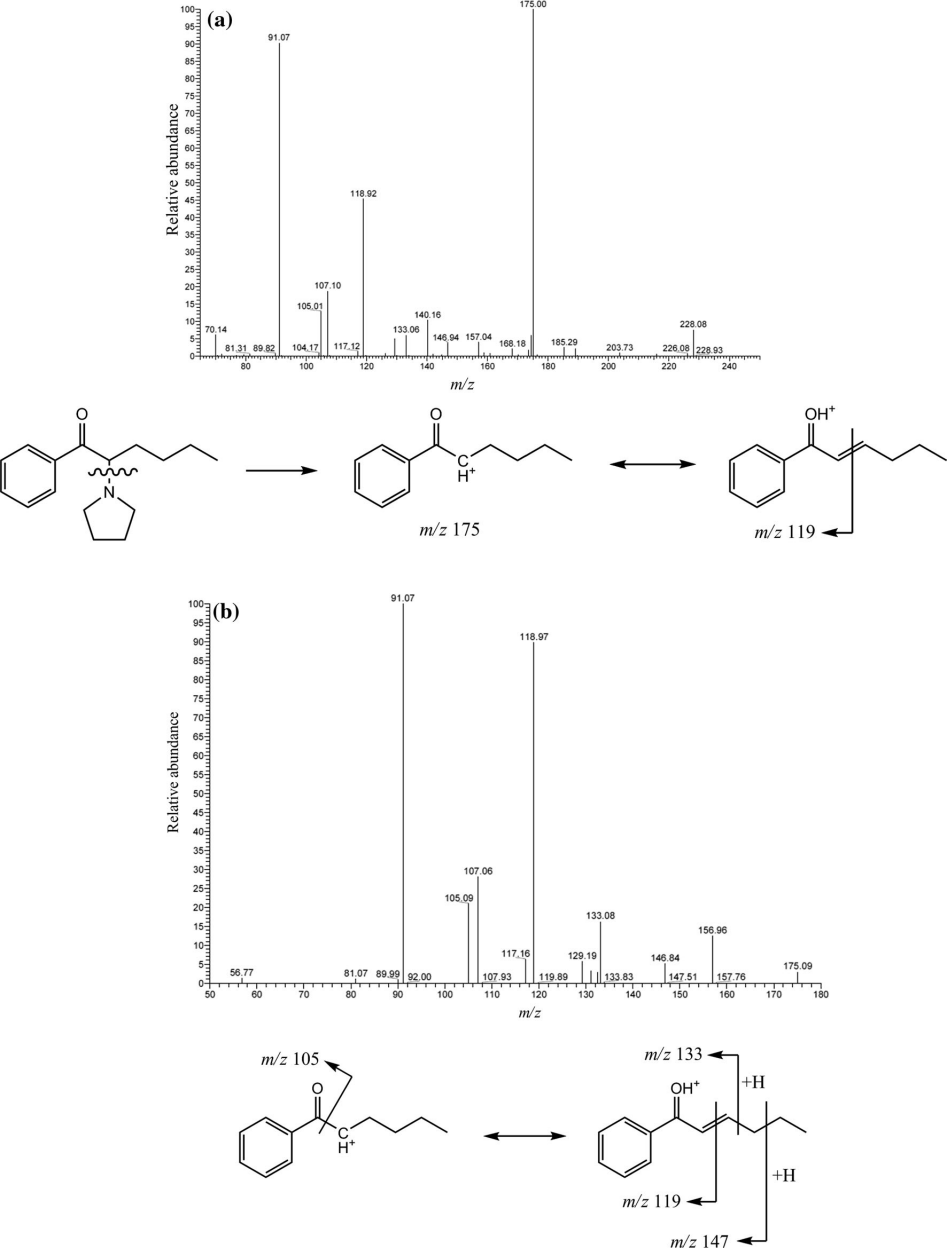
Product ion mass spectra obtained by ion trap MS in **a** MS^2^ (tandem) and **b** MS^3^ modes for sample no. 2. The precursor ions used for the MS^2^ and MS^3^ modes were those at *m/z* 246 and 175, respectively

Upon analysis of sample no. 2 by HPLC–MS, the TIC (Fig. 5a) showed an intense peak at 18.58 min. Otherwise, no marked peaks appeared, showing that sample no. 2 contained almost a single compound with high purity. Figure 5b shows the HPLC–MS spectrum of the intense peak observed in Fig. 5a; a base peak appeared at *m/z* 246. In the product ion mass spectrum in the MS^2^ mode (Fig. 6a), the peak due to elimination of one water molecule at *m/z* 228 also appeared, suggesting that the compound is also a cathinone derivative, and the peak due to the elimination of the pyrrolidinyl moiety appeared as the base peak at *m/z* 175. The product ion mass spectrum in the MS^3^ mode was also recorded using the ion at *m/z* 175 as the precursor ion for further analysis of the probable structure (Fig. 6b).

**Fig. 7:**
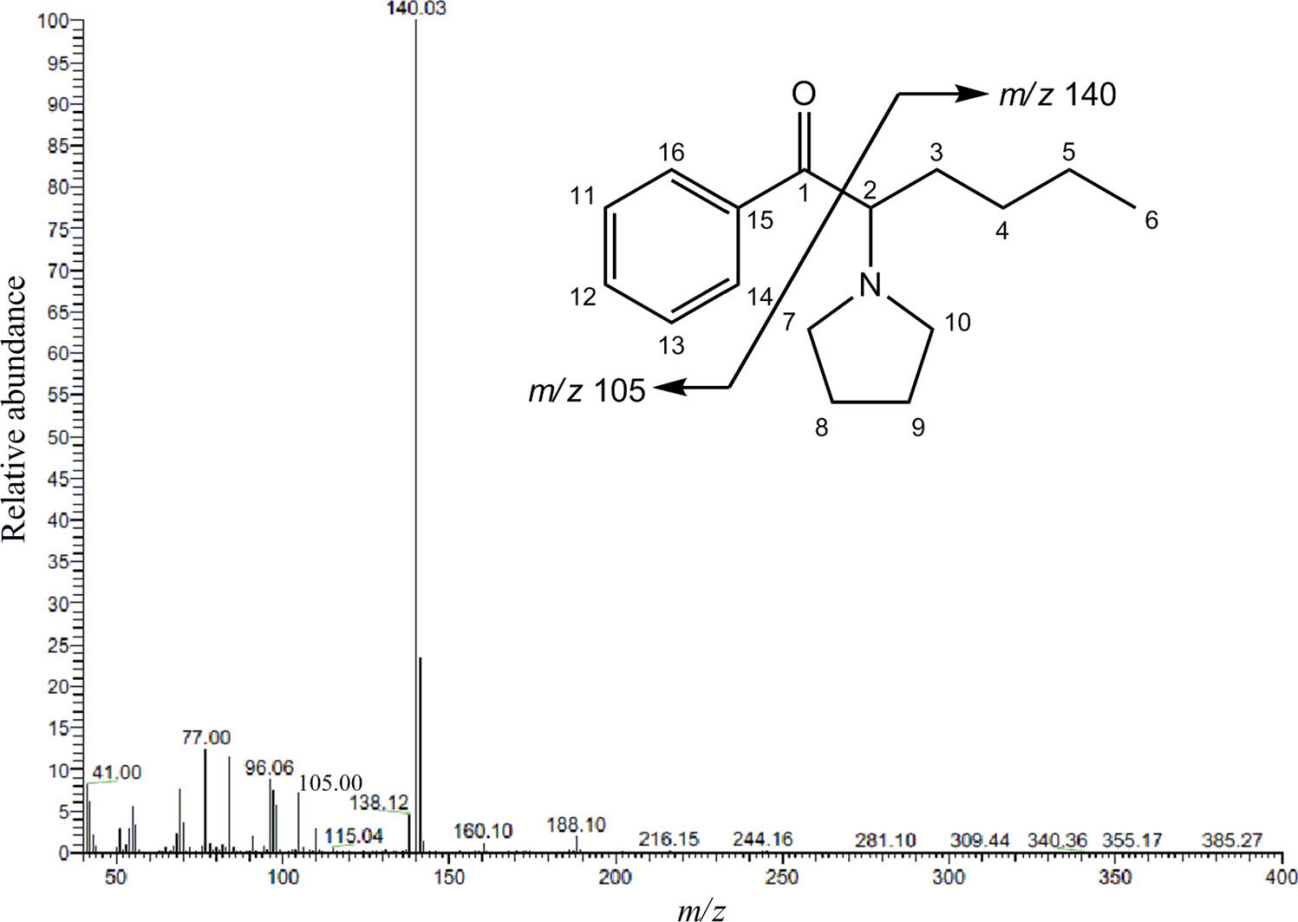
Mass spectrum of the compound contained in sample no. 2, obtained by GC–MS in the EI mode

Figure 7 shows the GC–MS spectrum of the compound in sample no. 2, and it was compared to that described in the library [28]. The EI-mass spectrum obtained from sample no. 2 was well matched that of α-PHP (1-phenyl-2-(1-pyrrolidinyl)-1-hexanone). In both spectra, the peak at *m/z* 140 appeared as the base peak; the fragment peaks at *m/z* 141, 105, 96 and 77 appeared in common.

As shown in Table 2, the data from ^1^H NMR and ^13^C NMR analysis of sample no. 2 confirmed the presence of α-PHP (the carbon numbering is shown in Fig. 4b). Similar to sample no. 1, the compound in sample no. 2 was also present in the hydrochloride form, which resulted in the appearance of a signal at 12.65 ppm originating from the proton of the quaternary pyrrolidine group.

**Table 2:** ^1^H and ^13^C NMR data for sample no. 2

Carbon atom position	Carbon chemical shifts (ppm)	Proton chemical shifts (ppm)
1	196.78	–
2	62.87	5.15 (dt, 1H)
3	30.62	2.03 (m, 2H)
4, 5	27.93; 22.50	1.33 (m, 4H)
6	13.48	0.84 (t, 3H)
7	52.90	2.83 (m, 1H); 3.68 (m, 1H)
8, 9	23.93; 23.71	2.21 (m, 4H)
10	49.51	3.84 (m, 2H)
11, 13	129.43	7.58 (t, 2H)
12	135.16	7.73 (t, 1H)
14,16	128.62	7.98 (d, 2H)
15	135.75	–

The carbon atom numbering is shown in Fig. 4b

It should be mentioned that Uchiyama et al. [27] reported the identification of α-PHP in an illegal product in 2014; they reported GC–MS mass spectrum, UV spectrum and ^1^H NMR and ^13^C NMR for α-PHP, which generally agree with the data described in this study.

### Other physicochemical analytical results for 4-fluoro-PV9 and α-PHP identified in this study

**Fig. 8:**
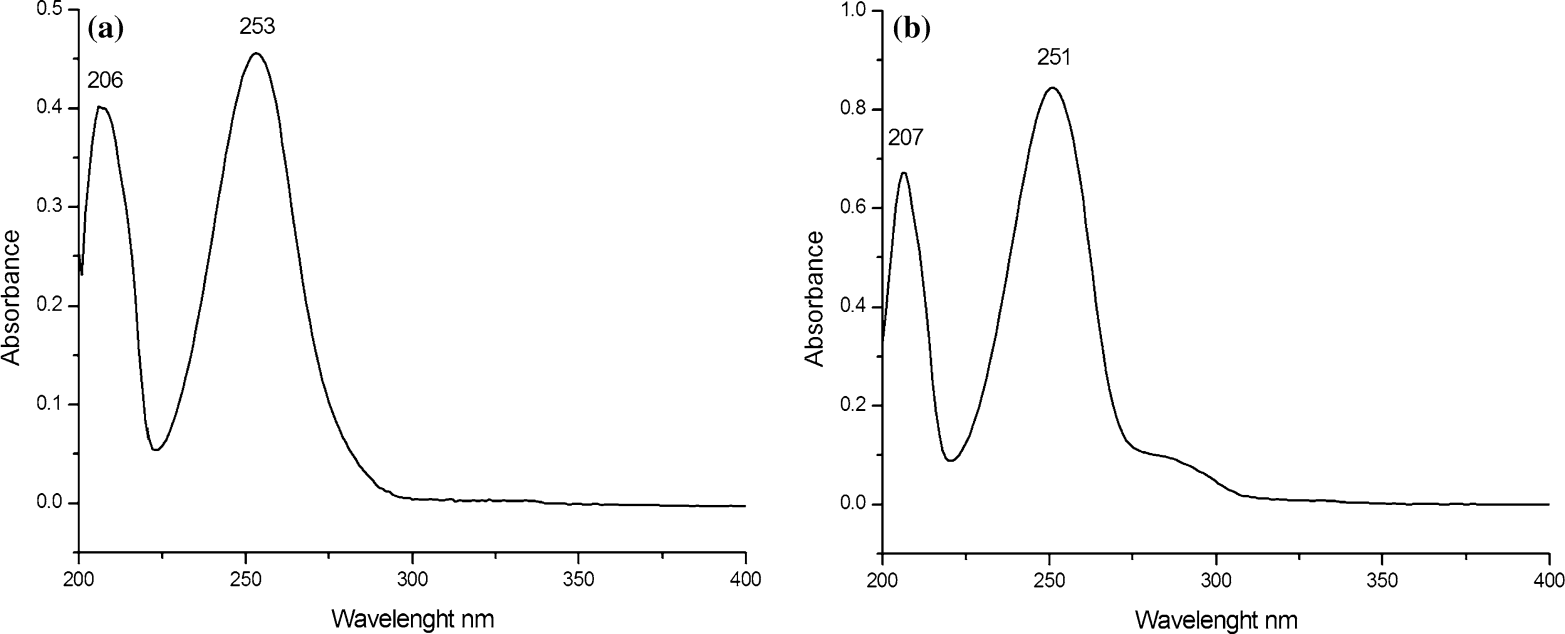
Ultraviolet-visible spectroscopy for the compounds contained in **a** sample no. 1 and **b** sample no. 2

The UV spectra recorded by HPLC–DAD and UV-VIS spectrometry showed absorption maxima at 254 and 253 nm, respectively, for the compound in sample no. 1 (4-fluoro-PV9); they were 252 and 251 nm, respectively, for that in sample no. 2 (α-PHP) (Fig. 8). The symmetrical peaks and the absence of impurity peaks in the spectra show that each compound existed in the materials in high purity.

**Fig. 9:**
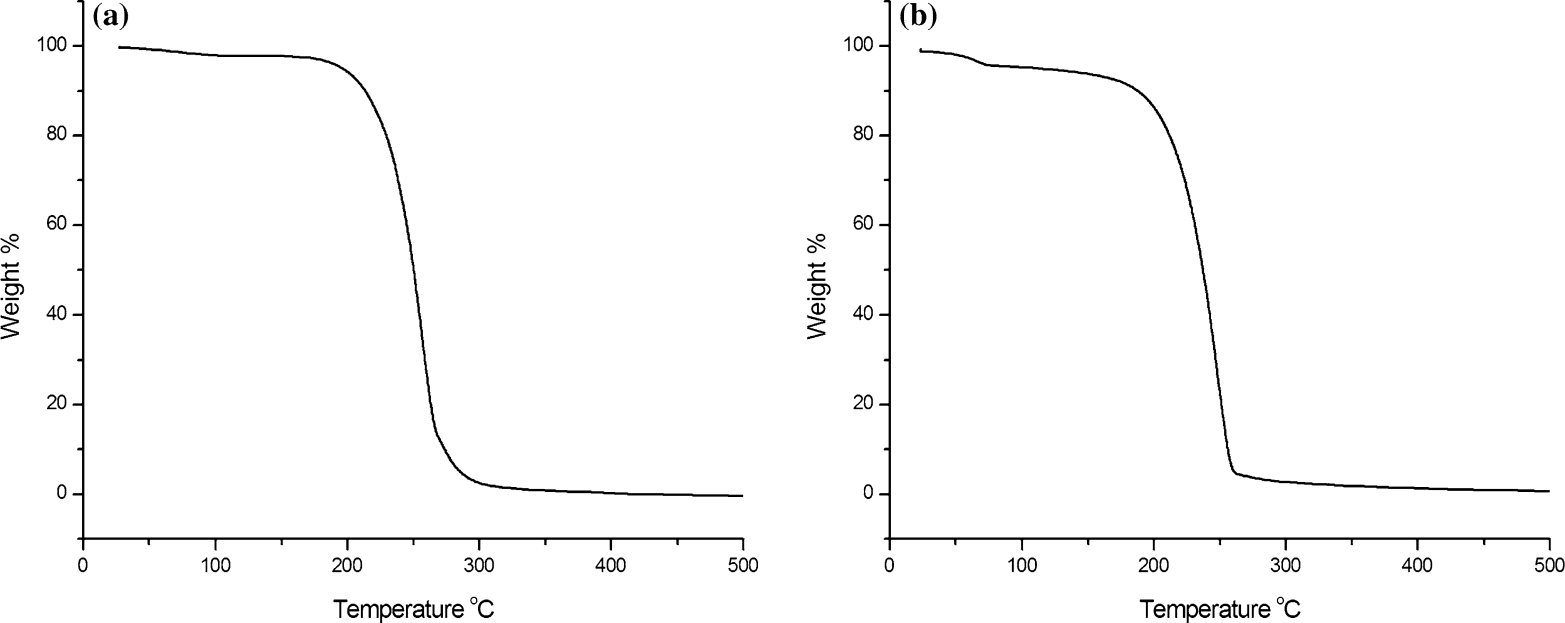
Thermogravimetric spectra for the compounds contained in **a** sample no. 1 and **b** sample no. 2

The TGA spectra for both compounds are shown in Fig. 9a, b, respectively. Both spectra showed single main transitions corresponding to their decomposition. The decomposition temperatures for those in sample no. 1 and sample no. 2 were 259 and 250 °C, respectively. The only one main transition observed for each compound shows that the purity of each compound was high.

By DSC analysis, melting points for the compounds in the samples no. 1 and no. 2 were 144 and 65 °C, respectively; those measured in the classic way were 140–142 and 62–64 °C, respectively.

By the FTIR spectroscopy, a strong carbonyl stretch at 1685 cm^−1^, the aliphatic CH stretching at 2800–3000 cm^−1^ and the amine hydrochloride salt bands at 2476–2752 cm^−1^ were observed for the compound in the sample no. 1; a strong carbonyl stretch at 1678 cm^−1^, the aliphatic CH stretching at 2800–3000 cm^−1^ and the amine hydrochloride salt bands at 2494–2750 cm^−1^ were observed for the compound in sample no. 2. Due to the structural similarity of both compounds, their IR spectra were very similar, as described above.

## Conclusions

In this study, we experienced the analysis of compounds in two seized materials. We used various analytical tools for their identification and characterization, such as HPLC–MS, HPLC–DAD, ion trap MS in MS^2^ and MS^3^ modes, GC–MS, TGA, DSC, FTIR spectroscopy, UV-VIS spectroscopy and ^1^H and ^13^C NMR spectroscopy. The compounds contained in sample no. 1 and no. 2 were found to be 4-fluoro-PV9 and α-PHP, respectively. Although α-PHP had been identified and characterized to some extent in 2014, to our knowledge, this is the first report on the identification and characterization of 4-fluoro-PV9. The data presented in this article will be useful for forensic toxicologists who are alert to the emergence of new psychotropic drugs.
